# Can different small-sided game formats impact physiological, physical, technical, and tactical demands in basketball players? A systematic review with meta-analysis

**DOI:** 10.5114/biolsport.2025.147012

**Published:** 2025-03-24

**Authors:** Tingyu Li, Shuang Wang, Diogo V. Martinho, Rui Miguel Silva, Qi Xu, Élvio R. Gouveia, Filipe Manuel Clemente

**Affiliations:** 1 Gdansk University of Physical Education and Sport, 80-336 Gdañsk, Poland; 2 Changsha Xiangjun Peicui Expermental Middle School, Changsha 410002, China; 3 University of Coimbra, Research Unit for Sport and Physical Activity, Faculty of Sport Sciences and Physical Education, Coimbra, Portugal; 4 Interactive Technologies Institute, Laboratory of Robotics and Engineering Systems, Funchal, Portugal; 5 Escola Superior Desporto e Lazer, Instituto Politécnico de Viana do Castelo, Rua Escola Industrial e Comercial de Nun’Álvares, 4900-347Viana do Castelo, Portugal; 6 Sport Physical Activity and Health Research & Innovation Center, Viana do Castelo, Portugal; 7 University of Madeira, Department of Physical Education and Sport, Funchal, Portugal

**Keywords:** Basketball, Conditioned games, Technical, Tactical

## Abstract

This systematic review with meta-analysis aimed to compare the effects of different game formats (1 v 1, 2 v 2, 3 v 3, 4 v 4, 4 v 3, 3 v 3+1, and 5 v 5) on basketball players’ physiological, physical, technical, and tactical responses during SSGs. The data sources utilized were PubMed, Scopus, SPORTDiscus, and Web of Science. Eligibility included basketball players of any age or sex, competing in tier 2 or higher, exposed to at least two different formats. Studies had to report on physiological responses, physical demands, technical performance, and tactical behaviors. Methodological quality was assessed using the MINORS scale. The search identified 4,967 titles, with 16 articles eligible for the review and meta-analysis. Results indicated that extreme SSGs (e.g., 1 v 1, 2 v 2) elicited significantly higher cardiovascular demands, as reflected by greater mean and peak heart rates, compared to larger SSGs (e.g., 3 v 3, 4 v 4), with a moderate effect size favoring extreme formats (Hedge’s g = -0.47, p = 0.02). In terms of perceived exertion (RPE), no significant differences were found between extreme and larger SSGs, suggesting similar subjective effort across formats. For technical performance, extreme SSGs (e.g., 1 v 1, 2 v 2) exhibited a higher frequency of actions, such as passes and shots, compared to larger formats, with a moderate effect size favoring smaller formats (Hedge’s g = -0.78, p < 0.01). No significant publication bias was found, though high heterogeneity was noted in RPE comparisons. This meta-analysis showed that extreme SSG formats elicit higher cardiovascular demands and more frequent technical actions than larger formats, highlighting their potential for targeting specific physical and technical demands in basketball training.

## INTRODUCTION

Small-sided games (SSGs) are drill-based activities frequently used by coaches to provide a multidimensional stimulus, integrating physical, technical, tactical, and cognitive demands to replicate the complexity of game scenarios and enhance player development [1]. In these games, the standard format and rules of a match are adjusted to align with the specific objectives of a training session [2]. These modifications help shape the playing dynamics, enhancing players’ awareness of specific collective and individual behaviors that ultimately influence their physiological, physical, technical, and tactical responses [3]. This approach allows coaches to modify players’ performance by manipulating various task constraints, such as playing area size, number of players, and specific rules, to achieve targeted objectives [4]. These games assume a relevant role within the training process as they replicate the physical, technical, tactical, and decision-making demands of the match [4].

Among the task constraints that coaches can manipulate, the playing format is one of the most commonly used and implemented [5]. The playing format is often defined by the number of players on each team and their numerical relationship with the opponent’s team. The play format in SSG is critical because it directly determines court dimensions, which vary depending on the number of players involved. For instance, smaller formats such as 1 v 1 or 2 v 2 typically use reduced court sizes to encourage close player interactions and frequent technical actions, while larger formats like 4 v 4 or 5 v 5 use proportionally larger areas to replicate full-game spatial dynamics and tactical complexity [6]. These variations in court dimensions ensure that the physical and tactical demands are appropriately matched to the number of participants. The number of players involved in the SSG and the court dimensions are often related. Therefore, to isolate the effect of the playing format (e.g., comparing 1 v 1 vs. 4 v 4 formats), the pitch dimensions are standardized to ensure the same player-to-square-meter ratio [7]. These two task constraints are the most studied and frequently implemented by coaches to regulate players’ performance.

Interest in studying the effects of manipulating different formats of play in basketball SSGs has grown in recent years [5]. Generally, the results suggest that extreme formats (e.g., 1 v 1 and 2 v 2) are associated with significantly higher heart rate responses and rates of perceived exertion [8]. This is likely due to the increased individual participation in the games, both at a tactical level—requiring players to engage more frequently in decision-making and spatial awareness— and at a technical level, with greater involvement in actions such as dribbling, passing, and shooting. This increased engagement reduces inactivity and rest time while elevating the frequency of metabolically demanding movements [9]. Additionally, some research indicates that smaller formats impact the number of individual technical actions performed by players [10, 11]. This is due to their participation without a clear tactical role, which impacts their involvement in both defensive and attacking moments. Furthermore, several studies have observed that the playing format promotes significant differences in the number of tactical solutions comprised of the decisions and actions executed by players to address game situations, such as positioning, passing options, and movement strategies [12].

Despite the existence of narrative [13] and systematic reviews [5] on the use of SSGs in basketball, which have addressed certain research questions, these reviews primarily summarized evidence without consolidating data through direct comparisons of game formats to examine their specific effects on players’ responses. Additionally, the only data that specifically addressed a research question about the effects of SSG-based programs on enhancing physical fitness [14]. Playing formats exemplify how task constraints can modulate performance, which is crucial for guiding coaches in selecting formats to achieve specific player responses. To answer such research questions, a systematic review with meta-analysis is the ideal approach. This method can summarize and provide solid evidence about the trends in results, clearly defining whether smaller or larger formats influence the intensity of physiological and physical demands or the frequency of technical actions and tactical requirements. Therefore, this systematic review with meta-analysis aimed to compare the effects of game formats on basketball players’ physiological, physical, technical, and tactical responses during SSGs.

## MATERIALS AND METHODS

Basketball is a dynamic sport that requires players to integrate technical, tactical, physical, and cognitive skills to succeed in competitive scenarios [15]. Different training methods often aim to replicate game conditions while targeting specific performance outcomes. Among these, small-sided games (SSGs) have emerged as a popular training method for their ability to simulate the demands of basketball [16]. However, a relevant question is commonly raised among coaches: *how do different SSG formats affect players’ physiological, physical, technical, and tactical responses?* Understanding these effects is essential for coaches aiming to optimize their training process and achieve greater levels of player adaptation. Yet, there is limited consensus regarding the relative benefits and trade-offs of smaller (e.g., 1 v 1, 2 v 2) versus larger (e.g., 3 v 3, 4 v 4) formats, leaving a gap in evidence-based practice [17].

This systematic review and meta-analysis adhered to the guidelines established by the Cochrane Collaboration and the PRISMA (Preferred Reporting Items for Systematic Reviews and Meta-analyses) framework [18]. Utilizing the PICOS methodology—Population, Intervention, Comparator, Outcomes, and Study Design—the review focused on basketball players of varying ages, sexes, and skill levels, excluding those with injuries, illnesses, or other medical conditions. The intervention examined smaller-sided game formats, while the comparator involved larger-sided game formats under similar experimental conditions. Outcomes measured included the mean and standard deviation (SD) values for key outcomes such as physiological responses, physical responses, technical actions, and tactical behaviors. The designs included were counterbalanced cross-over studies. This protocol was registered a priori on the OSF platform (https://doi.org/10.17605/OSF.IO/8QCZJ).

### Eligibility criteria

Details regarding the criteria for inclusion and exclusion in this systematic review and meta-analysis are outlined in Table 1. All original studies published in peer-reviewed journals or advance-of-print status were considered eligible for inclusion. No language restrictions were imposed on the articles included in this systematic review.

**TABLE 1:** Inclusion and exclusion criteria

Item	Inclusion criteria	Exclusion criteria
Population	Basketball players from any age-group, sex or skill level, e.g., tier 2 or above in the Participants Classification Framework [66], without injury, illness or other clinical condition.	Other sports than basketball (e.g., soccer, futsal or football indoor, beach soccer, American football, Australian football, handball, volleyball, hockey); parabasketball players, injured players, recreational-level players – tier 1).

Intervention	Smaller formats using any pitch dimension or other task condition. The following conditions were ensured: The same playing format was repeated at least two times (two repetitions) for the same players; The smaller format was extracted from the lowest format being compared (i.e., in case of studies comparing ≥ three formats for the same format or condition, only the smallest formats was extracted); The same experimental conditions between smaller and larger formats were ensured (i.e., same teams, same players, same time duration, same task constraints).	The same format was applied in only one repetition; Smaller and larger formats were not applied with same contextual and experimental conditions.

Comparator	Larger formats using any pitch dimension or other task condition. The following conditions were ensured: The same format was repeated at least two times (two repetitions) for the same players; The larger format was extracted (i.e., in case of studies comparing ≥ three formats for the same condition, only the largest format was extracted); The same experimental conditions between smaller and larger formats were ensured (i.e., same teams, same players, same time duration, same task constraints).	The same format was applied in only one repetition; Smaller and larger formats were not applied with same contextual and experimental conditions.

Outcome	At least one measure of the following possibilities: Physiological responses (e.g., heart rate, blood lactate concentrations or rated of perceived exertion); Physical demands (e.g., total distance, distances covered at different speed thresholds, acceleration/decelerations); Technical execution (e.g., passes, receptions, shots); Tactical behavior (e.g., attacking or defensive tactical principles, collective organization measures)	Other outcomes than those related to immediate physiological and physical, technical or tactical responses (e.g., fatigue tests, well-being tests).

Study design	A counterbalanced cross-over design.	Non-counterbalanced cross-over design studies.

Additional criteria	Peer reviewed, original, full-text studies written in English, Portuguese and/or Spanish.	Written in other language than those selected (English, Portuguese and/or Spanish). Reviews, letters to editors, trial registrations, proposals for protocols, editorials, book chapters, conference abstracts.

Two authors (TYL and DM) independently assessed the titles and abstracts of the retrieved records, followed by a separate independent review of the full texts. Any discrepancies among the authors were resolved through discussion and reevaluation. If a consensus could not be reached, a third reviewer (FMC) made the final determination. The EndNote X9.3.3 software was utilized for record management, including the automatic and manual removal of duplicates.

### Information sources

Electronic databases (PubMed, Scopus, SPORTDiscus, and Web of Science) were searched for relevant publications on 9 September 2024. To uncover additional relevant studies, we conducted a manual search of the reference lists from the included studies. We also employed snowball citation tracking using the Web of Science database. Additionally, each study included in the review was scrutinized for possible errors or retractions.

### Search strategy

The search was conducted using Boolean operators “AND” and “OR” without applying any filters for date, language, or study design, aiming to capture a broad spectrum of relevant studies. The detailed search approach was as follows:

[Title/Abstract] basket^*^

AND

[Title/Abstract] “small-sided games” OR “conditioned games” OR “SSG” OR “drill-based games” OR “small-sided conditioned games”

The full search strategy per database can be observed in Table 2.

**TABLE 2:** Full search strategy for each database.

PubMed	(((((basket* [Title/Abstract]) AND (“small-sided games” [Title/Abstract])) OR (“conditioned games” [Title/Abstract])) OR (“SSG” [Title/Abstract])) OR (“drill-based games” [Title/Abstract])) OR (“small-sided conditioned games” [Title/Abstract])

Scopus	(TITLE (basket*) OR ABS (basket*) AND TITLE (“small-sided games”) OR ABS (“small-sided games”) OR TITLE (“conditioned games”) OR ABS (“conditioned games”) OR TITLE (“SSG”) OR ABS (“SSG”) OR TITLE (“drill-based games”) OR ABS (“drill-based games”) OR TITLE (“small-sided conditioned games”) OR ABS (“small-sided conditioned games”))

SPORTDiscus	TI basket* AND TI “small-sided games” OR TI “conditioned games” OR TI “SSG” OR TI “drill-based games” OR TI “small-sided conditioned games”

Web of Science	((((((((((TI = (basket*)) OR AB = (basket*)) AND TI = (“small-sided games”)) OR AB = (“small-sided games”)) OR TI = (“conditioned games”)) OR AB = (“conditioned games”)) OR TI = (“SSG”)) OR AB = (“SSG”)) OR TI = (“drill-based games”)) OR AB = (“drill-based games”)) OR TI = (“small-sided conditioned games”)) OR AB = (“small-sided conditioned games”)

### Extraction of data

To evaluate inclusion criteria, a customized data extraction form inspired by the template from the Cochrane Consumers and Communication Review Group was employed. This form was initially trialed on ten randomly selected studies to ensure its effectiveness. Two independent reviewers (TYL and DM) carried out this assessment. Disputes over study inclusion were resolved through discussions among the reviewers and a third author (AFS). Reasons for excluding full-text articles were documented, and all records were systematically logged using a Microsoft Excel spreadsheet (Microsoft Corporation, Redmond, WA, USA).

### Data items

To ensure consistency in data analysis and reporting, only measures appearing in three or more studies were included. For physiological outcomes, the following outcomes were extracted: (i) cardiovascular responses (e.g., mean heart rate and peak heart rate), (ii) blood lactate levels, and (iii) rating of perceived exertion (RPE). For physical demands, the focus was on (i) total distance covered, (ii) distance traveled across various speed thresholds, (iii) number of accelerations and decelerations at different intensity levels, and (iv) mechanical workload metrics derived from inertial measurement units. In terms of technical performance, the metrics extracted included, for example, (i) total and accuracy-adjusted number of passes, (ii) total and accuracy-adjusted number of receptions, (iii) total and accuracy-adjusted number of shots, and (iv) total and accuracy-adjusted number of dribbles. For tactical behavior, we examined (i) individual attacking strategies, (ii) individual defensive strategies, and (iii) collective dispersion measures.

Data collected included sample size, mean, and standard deviation (SD) for each outcome in two different formats of SSG: extreme-sided games (ESG: 1 v 1; 2 v 2), medium-sided games (MSG: 3 v 3, 4 v 4), and 5 v 5. Further information extracted from the studies comprised: (i) participant details (number, age, competitive level, and sex); (ii) outcomes examined (physical, physiological, technical, and tactical), (iii) variables derived from each outcome; (iii) SSG format (e.g., 1 v 1, 2 v 2, 3 v 3, and 4 v 4) and 5 v 5, pitch dimensions, and relative area per player; (iv) specific rules. Where data were shown graphically, a specific software was used to extract mean and standard deviation (http://www.getdata-graph-digitizer.com). This software is accurate and precise in extracting these parameters [19].

### Risk of bias assessment

The assessment of methodological quality for the included studies was conducted using the Methodological Index for Non-Randomized Studies (MINORS). This validated tool evaluates twelve specific criteria from the studies, with a scoring system where zero denotes no report on the criterion, one signifies an inadequate report, and two indicates an adequate report. In this review, we focused on eight key items from the MINORS tool: (1) a clearly stated aim, (2) inclusion of consecutive patients, (3) prospective collection of data, (4) endpoints appropriate to the aim of the study, (5) unbiased assessment of the study endpoint, (6) an appropriate follow-up period, (7) loss to follow-up of less than 5%, and (8) prospective calculation of study size. The total score for each study was calculated by summing the individual scores across these eight criteria, yielding a maximum possible score of 16 points. To categorize the studies based on their overall methodological quality, studies were classified as good, fair, or poor, depending on their total score. A score between 14 and 16 points was considered good, indicating a high methodological quality. A score between 10 and 13 points was classified as fair, suggesting moderate methodological quality. Studies with a score below ten were categorized as poor. Two reviewers (TYL and RMS) independently applied this scale to assess the quality of the studies. Discrepancies in ratings were addressed through discussions, with a final resolution provided by a third reviewer (FMC).

### Summary measures, synthesis of results, and publication bias

Meta-analysis typically requires at least two studies to generate valid results [20], but due to the frequent small sample sizes in sports science research [21], and especially in SSG studies [22], this meta-analysis only included data if at least three study groups provided both means and standard deviations for comparisons of different game formats of a specific variable. Comparisons were performed considering three groups: extreme-sided games (1 v 1, 2 v 2), medium-sided games (3v3, 4v4), and 5v5. Effect sizes (ES; Hedge’s g) were computed for each outcome using these means and SDs. When means and SDs were not directly available, they were derived from 95% confidence intervals (CIs) or standard errors of the mean (SEM), utilizing Cochrane’s RevMan Calculator. A reductionist approach was chosen to combine multiple effect sizes of the same study [23]

A random-effects model was applied to accommodate variability between studies that could affect the SSG outcomes [24, 25]. Effect sizes are reported with 95% confidence intervals. The ES values were classified according to the following scale: < 0.2 as trivial, 0.2–0.6 as small, > 0.6–1.2 as moderate, > 1.2–2.0 as large, > 2.0–4.0 as very large, and > 4.0 as extremely large [26]. Heterogeneity was evaluated using the I^2^ statistic, with thresholds of < 25%, 25–75%, and > 75% indicating low, moderate, and high heterogeneity, respectively [27]. The risk of bias was assessed using the extended Egger’s test [28]. To address potential publication bias, a sensitivity analysis was performed employing the trim and fill method [29]. All statistical analyses were conducted with Comprehensive Meta-Analysis software (version 2; Biostat, Englewood, NJ, USA), with statistical significance set at *p* < 0.05.

## RESULTS

### Study identification and selection

**FIG. 1:**
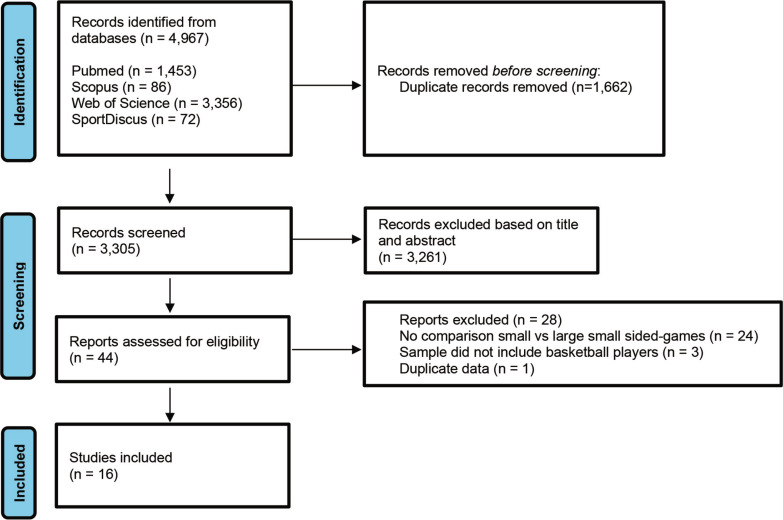
Flow chart diagram identifying the screening process and the studies included in the present scoping review.

An initial search was conducted across four electronic databases, yielding a total of 4,967 records, of which, after removing 1,661 duplicates, 3,306 records were screened based on their titles and abstracts. From this screening process, 3,261 records were excluded, and the full texts of 44 manuscripts were analyzed in detail. Twenty-eight papers were excluded based on specific criteria: (i) 24 studies did not compare different types of small-sided games; (ii) 3 studies had samples that did not include basketball players; (iii) one study presented duplicate data. As a result, this review includes a total of 16 studies, as illustrated in Figure 1.

### Risk of bias assessment

The MINORS assessment for risk of bias (table 3) across the included studies revealed that most studies scored well in defining clear aims, including consecutive participants, collecting data prospectively, aligning endpoints with study objectives, and ensuring unbiased assessment of these endpoints, with scores typically at 2 out of 2 for these criteria. However, there is a greater variability and lower scores in the follow-up period’s appropriateness loss to follow-up, particularly regarding the prospective calculation of sample size, where the overall studies scored 0 due to the absence of sample size calculations. This may indicate a potential risk of bias related to sample size adequacy and the representativeness of the follow-up data. However, the overall scores ranging from 9 to 14 out of 16 showed that the included studies have a moderate to good methodological quality.

**TABLE 3:** MINORS Risk of Bias assessment.

	Item 1	Item 2	Item 3	Item 4	Item 5	Item 6	Item 7	Item 8	Total
Sansone et al. [30]	2	2	2	2	2	2	2	0	14 / 16
Gok et al. [41]	2	2	2	2	2	1	0	0	11 / 16
Bredt et al. [31]	2	2	2	2	2	0	0	0	10 / 16
Bredt et al. [40]	2	2	2	2	2	0	0	0	10 / 16
Stojanovic et al. [56]	2	2	2	2	2	0	0	0	10 / 16
Clemente et al. [42]	2	2	2	2	2	0	0	0	10 / 16
Ferioli et al. [33]	2	2	2	2	2	2	2	0	14 / 16
Clemente et al. [34]	2	2	2	2	2	2	0	0	12 / 16
Vaquera et al. [35]	2	2	2	2	2	2	0	0	12 / 16
Conte et al. [36]	2	2	2	2	2	2	0	0	12 / 16
Herran et al. [43]	2	2	2	2	1	1	0	0	10 / 16
Conte et al. [37]	2	2	2	2	2	2	0	0	12 / 16
Garcia et al. [38]	1	1	2	2	2	1	1	0	10 / 16
Klusemann et al. [45]	2	1	2	2	1	1	1	0	10 / 16
Tallir et al. [44]	2	1	2	2	1	1	0	0	10 / 16
Sampaio et al. [39]	2	1	2	2	1	1	0	0	9 / 16

Item 1: A clearly stated aim; Item 2: Inclusion of consecutive patients; Item 3: Prospective collection of data; Item 4: Endpoints appropriate to the aim of the study; Item 5: Unbiased assessment of the study endpoint; Item 6: Follow-up period appropriate to the aim of the study; Item 7: Loss to follow up less than 5%; Item 8: Prospective calculation of the study size.

### Study characteristics

**FIG. 2:**
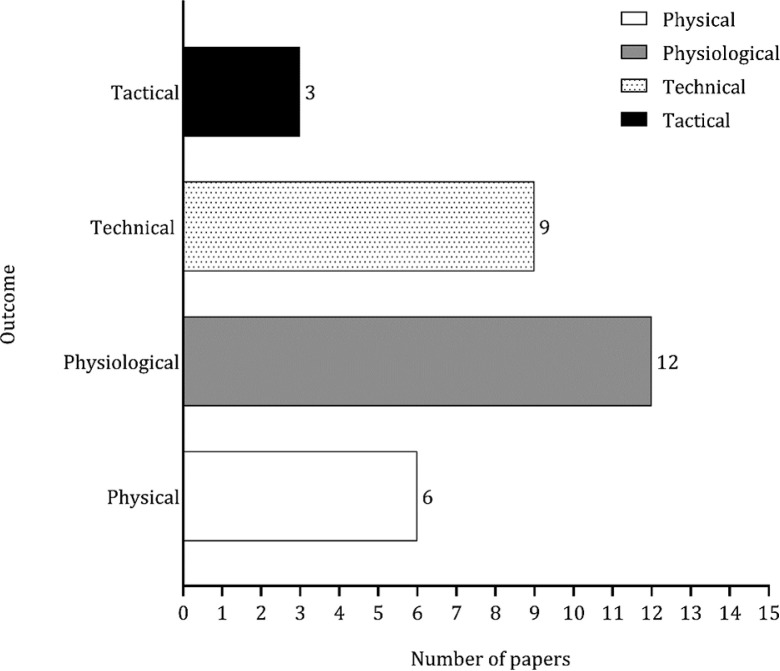
Main outcomes analyzed.

**FIG. 3:**
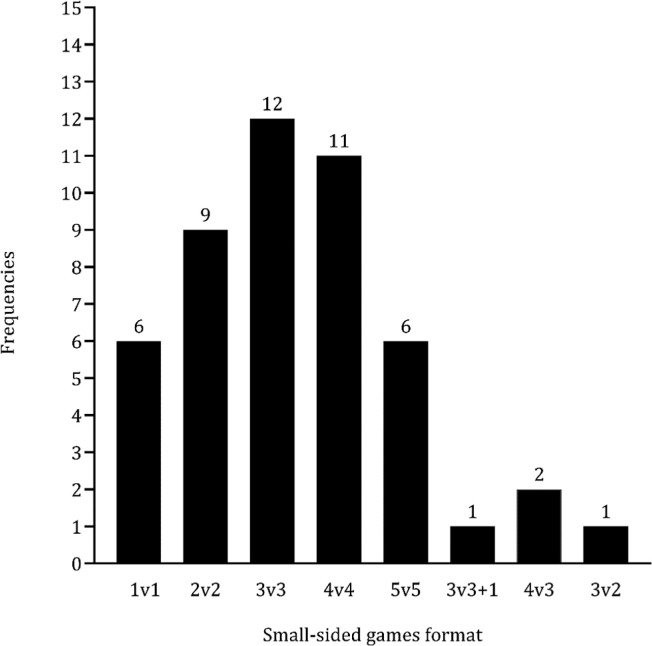
Main formats of play analyzed.

Table 4 summarizes the key information obtained from each study, including the country of origin, competitive level, sample size, age of participants, outcomes analyzed, and associated variables. A total of 309 basketball players were included in this systematic review. Eleven studies focused on men [30–40], while three studies investigated female athletes [41–43]. Two studies examined both sexes [44, 45]. Physiological outcomes were examined in 12 studies (approximately 71%), while tactical and physical outcomes were represented in 9 (approximately 56%) and 6 (approximately 38%) studies, respectively. Three studies focused on tactical outcomes, as shown in Figure 2. The review covered a total of 15 studies on extreme-sided games and 33 studies on medium-sided games. The comparison between game formats also considered additional variations (e.g., 3 v 3+1, 4 v 3, and 3 v 2), as illustrated in Figure 3.

**TABLE 4:** Characteristics of the studies included in the present review, outcomes examined, and variables assessed.

Study	Country	Competitive level	Sample size (n)	Sex	Age (years)	Outcome	Variable extracted
Sansone et al. [30]	Italy	Regional	15	M	15.6 ± 0.7	Physical	Average external load intensity, peak external load intensity

Gok et al. [41]	NI	Regional	18	F	15.6 ± 0.7	Physiological	Rate of perceived exertion

Gok et al. [41]						Technical	Rebound, successful shot, unsuccessful shot, successful pass, unsuccessful pass, steal, turnover

Bredt et al. [31]	NI	National	51	M	Under-14: 13.7 ± 0.3 Under-15: 14.7 ± 0.3 Under-15: 14.7 ± 0.3	Tactical	Space creation with ball dribbled, space creation with ball not dribbled, perimeter isolation, poster isolation, space creation without ball, on-ball screen, offenses with no shots, close on-ball marking, close on-ball marking, fluctuated on-ball marking, close off-ball marking, fluctuated off-ball marking, defensive help or switch, defensive recovery, double-team

Bredt et al. [31]						Technical	number of passes per offense, total number of offenses, closeout, steal, defensive rebound

Bredt et al. [40]	NI	National	45	M	Under-14: 13.7 ± 0.3 Under-15: 14.7 ± 0.3 Under-15: 14.7 ± 0.3	Physical	Acceleration

Bredt et al. [40]						Physiological	Heart rate

Bredt et al. [40]						Tactical	Space creation with ball dribbled, perimeter isolation, space creation without ball, total number of offenses, fast-breaks, offenses with no shot, participation of floater, floater, number of passes per offense

Stojanovic et al. [56]	NI	Recreational	12	M	21.4 ± 1.7	Physical	Total distance, acceleration, deceleration

Stojanovic et al. [56]						Physiological	Heart rate, blood lactate, rate of perceived exertion

Clemente et al. [42]	NI	National	10	F	14.3 ± 0.3	Physiological	Rate of perceived exertion

Clemente et al. [42]						Technical	Received balls, conquered balls, lost balls, attacking balls, rebounds, shots

Ferioli et al. [33]	Italy	Regional	10	M	18.3 ± 1.0	Physical	Low-intensity activities, medium-intensity activities, high-intensity activities, recovery, all movements

Ferioli et al. [33]						Physiological	Heart rate, rate of perceived exertion

Ferioli et al. [33]						Technical	Assists, personal fouls, rebounds, steals, turnovers, total passes, correct passes, shots, scored shots

Clemente et al. [34]	NI	National	20	M	Under-14: 12.0 ± 0.8 Under-16: 14.3 ± 0.5	Physiological	Rate of perceived exertion

Clemente et al. [34]						Technical	Received balls, conquered balls, lost balls, attacking balls, shot, rebound

Vaquera et al. [35]	Spain	Junior	12	M	16.0 ± 0.4	Physiological	Heart rate, rate of perceived exertion

Conte et al. [36]	Italy	Regional	12	M	13.9 ± 0.7	Physiological	Heart rate, rate of perceived exertion

Conte et al. [36]						Technical	Dribble, pass, shot, interception, steal, rebound, turnover

Herran et al. [43]	NI	Junior	10	F	15.1 ± 1.0	Physical	Total distance, player load, maximum velocity, velocity, acceleration, deceleration

Conte et al. [37]	NI	Regional	21	M	15.4 ± 0.9	Physiological	Heart rate, rate of perceived exertion, Edwards’ TRIMP

Conte et al. [37]						Technical	Dribble, steal, rebound, turnover, pass, shot

Garcia et al. [38]	Spain	National	19	M	14.5 ± 1.5	Physiological	Heart rate

Klusemann et al. [45]	Australia	Elite	16	8 M; 8 F	M: 18.2 ± 0.6 F: 17.4 ± 0.3	Physical	Stand/walk, jog, run, sprint, low shuffle, medium shuffle, high shuffle, jump, total movements

Klusemann et al. [45]						Physiological	Heart rate, rate of perceived exertion

Klusemann et al. [45]						Technical	Dribble, pass, close range shot, mid-range shot, 3-point shot, rebound, ball screen, total elements

Tallir et al. [44]	Belgium	Junior	30	23 M; 7 F	11.1 ± 0.6	Tactical	Cognitive decision-making component

Tallir et al. [44]						Technical	Motor skill execution efficiency, motor skill execution efficacy

Sampaio et al. [39]	Portugal	NI	8	M	15.5 ± 0.6	Physiological	Heart rate, rate of perceived exertion

NI (no information); M (male); F (female)

Table 5 presents the descriptive statistics for the comparisons between different basketball formats. Due to the numerous variables extracted from the four main outcomes — physical, physiological, technical, and tactical — a meta-analysis was conducted specifically for the rate of perceived exertion, cardiovascular demands (including peak heart rate, mean heart rate, and heart rate at specific intensities), and technical parameters (such as dribbling, passing, shooting, rebounds, assists, and steals).

**TABLE 5:** Characteristics of small-sided games in the included studies.

Study	SSG formats	Court dimension (m) | Ratio area/player (m^2^)	SSG exercise characteristics	Rules
Sansone et al. [30]	1 v 1, 2 v 2, 3 v 3, 4 v 4, 5 v 5, 5 v 5 scrimmage	NI	NI	5 v 5 scrimmage: competition rules

Gok et al. [41]	2 v 2, 3 v 3, 4 v 4	28.0 × 15.0 | NI	4 × 4 min /4 min recovery	NI

Bredt et al. [31]	3 v 3, 4 v 3	3 v 3: 14.0 × 9.0 | 42 4 v 3: 14.0 × 9.0 | 31.5	1 × 4 min /8 min recovery	No free throws

Bredt et al. [40]	3 v 3, 3 v 3+1, 4 v 3	3 v 3: 28.0 × 9.0 | 84 4 v 3: 28.0 × 9.0 | 63	1 × 4 min /8 min recovery	No free throws

Stojanovic et al. [56]	1 v 1, 2 v 2, 3 v 3	1 v 1: 14.0 × 15.0 | 210 2 v 2: 14.0 × 15.0 | 105 3 v 3: 14.0 × 15.0 | 70	1 × 10 min /5 min recovery	No free throws, after a defensive rebound the ball needs to be played out from the

three-point line

Clemente et al. [42]	1 v 1, 2 v 2, 3 v 3, 4 v 4, 5 v 5	1 v 1: 15.0 × 6.0 | 45 2v 2: 22.0 × 8.0 | 44 3v 3: 24.0 × 11.0 | 44 4v 4: 26.0 × 13.0 | 42 5v 5: 28.0 × 15.0 | 42	1 v 1: 2 × 1 min /2 min recovery 2 v 2: 2 × 2 min / 2 min recovery 3 v 3: 2 × 3 min / 2 min recovery 4 v 4: 2 × 4 min / 2 min recovery 5 v 5: 2 × 5 min / 2 min recovery	No free throws

Ferioli et al. [33]	3 v 3, 4 v 4, 5 v 5	3 v 3: 28.0 × 15.0 | 70 4 v 4: 28.0 × 15.0 | 52.5 5 v 5: 28.0 × 15.0 | 42	3 × 4 min /2 min recovery	No free throws, throw-ins after a foul, one-on-one defence

Clemente et al. [34]	1 v 1, 2 v 2, 3 v 3, 4 v 4, 5 v 5	1 v 1: 15.0 × 6.0 | 45 2 v 2: 22.0 × 8.0 | 44 3 v 3: 24.0 × 11.0 | 44 4 v 4: 26.0 × 13.0 | 42 5 v 5: 28.0 × 15.0 | 42	1 v 1: 2 × 1 min /2 min recovery 2 v 2: 2 × 2 min / 2 min recovery 3 v 3: 2 × 3 min / 2 min recovery 4 v 4: 2 × 4 min / 2 min recovery 5 v 5: 2 × 5 min / 2 min recovery	No free throws

Vaquera et al. [35]	1 v 1, 2 v 2, 3 v 2, 5 v 5	28.0 × 15.0 | NI	2–3 minutes recovery	One-on-one defence, no free-throws

Conte et al. [36]	2 v 2, 4 v 4	2 v 2: 28.0 × 15.0 | 105	3 × 4 min / 2 min recovery	One-on-one defence

Herran et al. [43]	3 v 3, 5 v 5	5 v 5: 15.0 × 14.0 | 21	1 × 5 min / 5 min recovery	No fouls, static position in out of bonds, after a successful shoot the further player make the ball playable, after a defensive rebound the ball needs to be played out from the three-point line via passing

Conte et al. [37]	2 v 2, 4 v 4	2 v 2: 28.0 × 15.0 | 105 4 v 4: 28.0 × 15.0 | 52.5	CONT: 3 × 4 min / 2 min passive recovery INTER: 3 × 7 min / 1 min exercise / 1 min passive rest	One-on-one defence

Garcia et al. [38]	3 v 3, 4 v 4	3 v 3: 14.0 × 15.0 | 35	4 min / 3 min recovery	NI

Klusemann et al. [45]	2 v 2, 4 v 4	2 v 2: 15.0 × 14.0 | 52.5 4 v 4: 15.0 × 14.0 | 26.3	NI	12 second shot clock, reward a point when the foul is committed in shooting

Tallir et al. [44]	3 v 3, 4 v 4	4 v 4: 14.0 × 26.0 | 30.3	Smaller: 2 or 3 4 × 5 min / 5 min recovery Larger: 4 × 5 min / 5 min recovery	One-on-one defence

Sampaio et al. [39]	3 v 3, 4 v 4	3 v 3: 14.7 × 4.9 | 12 4 v 4: 19.8 × 6.6 | 16.8	4 × 4 min / 3 min recovery	NI

SSG (small-sided games); NI (no information); CONT (continuous exercise) INTER (intermittent exercise).

**TABLE 6:** Comparisons regarding different formats of small-sided games on physical, physiological, technical or tactical outcomes.

Study	Game formats		Variable extracted (unit)	Mean ± SD		Included meta-analysis
	Format 1	Format 2		Format 1	Format 2	
Sansone et al. [30]	1 v 1, 2 v 2	3 v 3, 4 v 4	Peak external load (peak EL • min^–1^)	178 ± 23	183 ± 22	No
			Average external load (peak EL • min^–1^)	301 ± 39	354 ± 38	No
	3 v 3, 4 v 4	5 v 5	Peak external load (peak EL • min^–1^)	183 ± 22	183 ± 21	No
			Average external load (peak EL • min^–1^)	354 ± 38	372 ± 5	No
	1 v 1, 2 v 2	5 v 5	Peak external load (peak EL • min^–1^)	178 ± 23	183 ± 21	No
			Average external load (peak EL • min^–1^)	301 ± 39	372 ± 5	No

Gok et al. [41]	2 v 2	3 v 3, 4 v 4	RPE (AU)	14.5 ± 1.1	12.9 ± 1.9	Yes
			Rebound (count)	0.9 ± 0.3	0.7 ± 0.3	Yes
			Successful shot (count)	1.9 ± 1.1	1.3 ± 0.4	Yes
			Unsuccessful shot (count)	2.1 ± 0.7	1.6 ± 0.5	No
			Successful pass count)	3.7 ± 1.5	4.0 ± 1.0	Yes
			Unsuccessful pass (count)	0.3 ± 0.2	0.4 ± 0.2	No
			Stealing (count)	0.2 ± 0.2	0.4 ± 0.3	Yes
			Turnover (count)	0.4 ± 0.3	0.6 ± 0.4	No

Bredt et al. [31]	3 v 3	4 v 3	Acceleration 0.0–0.5 g (seconds)	168 ± 21	182 ± 13	No
			Acceleration 0.5–1.0 g (seconds)	63 ± 22	49 ± 14	No
			Acceleration 1.0–1.5 g (seconds)	7.2 ± 2.3	6.5 ± 2.5	No
			Acceleration 1.5–2.0 g (seconds)	1.1 ± 0.7	1.1 ± 0.9	No
			Mean HR (% of maximal)	86.2 ± 3.3	85.9 ± 4.9	No
			Peak HR (% of maximal)	93.6 ± 3.2	92.0 ± 4.0	No
			HR > 85% (seconds)	158.7 ± 53.6	148.8 ± 63.4	No
			Space creation with ball dribbled (count)^¶^	6.0	5.5	No
			Space creation with ball not dribbled (count) ^¶^	1.0	0.0	No
			Perimeter isolation (count) ^¶^	3.0	1.5	No
			Post isolation (count) ^¶^	0.5	0.0	No
			Space creation without ball (count) ^¶^	7.0	2.5	No
			On-ball screen (count) ^¶^	0.0	0.0	No
			Passes per offense (count) ^¶^	1.57	2.65	No
			Total number of offenses (count) ^¶^	22.5	23.5	No
			Offenses with no shot (count) ^¶^	3.0	2.5	No
			Close on-ball marking (count) ^¶^	3.0	2.0	No
			Fluctuated on-ball marking (count) ^¶^	4.0	2.0	No
			Close off-ball marking (count) ^¶^	3.5	3.0	No
			Fluctuated off-ball marking (count) ^¶^	8.0	8.0	No
			Defensive help or switch (count) ^¶^	2.0	1.0	No
			Closeout (n) ^¶^	2.0	6.0	No
			Double-team (n) ^¶^	0.5	0.0	No
			Steal/ interception (n) ^¶^	0.0	0.0	No
			Defensive rebound (n) ^¶^	1.0	1.0	No

Bredt et al. [40]	3 v 3	4 v 3	Acceleration 0.0–0.5 g (seconds)	144 ± 24	153 ± 24	No
			Acceleration 0.5–1.0 g (seconds)	64 ± 44	69 ± 34	No
			Acceleration 1.0–1.5 g (seconds)	7.0 ± 2.9	6.0 ± 2.5	No
			Acceleration 1.5–2.0 g (seconds)	1.0 ± 0.7	1.0 ± 0.6	No
			Mean HR (% of maximal)	86.5 ± 3.6	85.0 ± 4.1	No
			Peak HR (% of maximal)	93.8 ± 3.2	92.2 ± 4.2	No
			HR > 85% (time)	158 ± 55	138 ± 67	No
			Space creation with ball dribbled (count) ^¶^	7.0	7.0	No
			Perimeter isolation (count) ^¶^	1.5	0.5	No
			Space creation without ball (count) ^¶^	7.5	12.5	No
			Total number of offenses (count) ^¶^	22.0	23.0	No
			Fast-breaks (count) ^¶^	6.0	7.0	No
			Offenses with no shot (count) ^¶^	3.0	3.0	No
			Participation of floater (count) ^¶^	NI	10.5	No
			Decisive floater (count) ^¶^	NI	8.5	No
			Passes per offense (count) ^¶^	1.62	2.1	No
	3 v 3	3 v 3+1	Acceleration 0.0–0.5 g (seconds)	144 ± 24	150 ± 21	No
			Acceleration 0.5–1.0 g (seconds)	64 ± 44	82 ± 24	No
			Acceleration 1.0–1.5 g (seconds)	7.0 ± 2.9	6.2 ± 2.6	No
			Acceleration 1.5–2.0 g (seconds)	1.0 ± 0.7	1.2 ± 0.7	No
			Mean HR (% of maximal)	86.5 ± 3.6	84.9 ± 3.5	No
			Peak HR (% of maximal)	93.8 ± 3.2	92.9 ± 3.7	No
			HR > 85% (time)	158 ± 55	135 ± 74	No
			Space creation with ball dribbled (count) ^¶^	7.0	5.0	No
			Perimeter isolation (count) ^¶^	1.5	1.0	No
			Space creation without ball (count) ^¶^	7.5	9.0	No
			Total number of offenses (count) ^¶^	22.0	20.5	No
			Fast-breaks (count) ^¶^	6.0	7.0	No
			Offenses with no shot (count) ^¶^	3.0	3.0	No
			Participation of floater (count) ^¶^	NI	10.5	No
			Decisive floater (count) ^¶^	NI	8.5	No
			Passes per offense (count) ^¶^	1.62	2.1	No

Stojanovic et al. [56]	1 v 1, 2 v 2	3 v 3	Total distance (m)	715 ± 180	673 ± 181	No
			Acceleration (count)	165 ± 15	145 ± 16	No
			Deceleration (count)	165 ± 15	144 ± 16	No
			Mean HR (bpm)	179 ± 9	174 ± 9	Yes
			Maximal HR (bpm)	193 ± 6	190 ± 7	Yes
			Mean HR (% of maximal)	88.1 ± 5.5	86.1 ± 5.8	Yes
			Maximal HR (% of maximal)	95.1 ± 4.7	93.2 ± 5.7	Yes
			Blood lactate (mmol • L–1)	5.0 ± 2.1	4.0 ± 1.2	No
			RPE (AU)	5.3 ± 1.6	4.1 ± 1.5	No

Clemente et al. [42]	1 v 1, 2 v 2	3 v 3, 4 v 4	RPE (AU)	3.7 ± 1.0	5.2 ± 1.7	Yes
			Received balls (count • min^–1^)	1.6 ± 0.9	1.2 ± 0.5	Yes
			Conquered balls (count • min^–1^)	0.3 ± 0.4	0.2 ± 0.2	Yes
			Lost balls (count • min^–1^)	0.5 ± 0.5	0.2 ± 0.2	No
			Attacking balls (count • min^–1^)	1.0 ± 0.6	0.6 ± 0.6	Yes
			Shot (count • min^–1^)	2.0 ± 0.9	0.7 ± 0.5	Yes
			Rebound (count • min^–1^)	1.0 ± 0.6	0.3 ± 0.3	Yes
	3 v 3, 4 v 4	5 v 5	RPE (AU)	5.2 ± 1.7	7.2 ± 1.3	Yes
			Received balls (count • min^–1^)	1.2 ± 0.5	1.1 ± 0.5	Yes
			Conquered balls (count • min^–1^)	0.2 ± 0.2	0.2 ± 0.2	Yes
			Lost balls (count • min^–1^)	0.2 ± 0.2	0.2 ± 0.2	No
			Attacking balls (count • min^–1^)	0.6 ± 0.6	0.6 ± 0.4	Yes
			Shot (count • min^–1^)	0.7 ± 0.5	0.4 ± 0.3	Yes
			Rebound (count • min^–1^)	0.3 ± 0.3	0.2 ± 0.2	Yes
	1 v 1, 2 v 2	5 v 5	RPE (AU)	3.7 ± 1.0	7.2 ± 1.3	Yes
			Received balls (count • min^–1^)	1.6 ± 0.9	1.1 ± 0.5	No
			Conquered balls (count • min^–1^)	0.3 ± 0.4	0.2 ± 0.2	No
			Lost balls (count • min^–1^)	0.5 ± 0.5	0.2 ± 0.2	No
			Attacking balls (count • min^–1^)	1.0 ± 0.6	0.6 ± 0.4	No
			Shot (count • min^–1^)	2.0 ± 0.9	0.4 ± 0.3	No
			Rebound (count • min^–1^)	1.0 ± 0.6	0.2 ± 0.2	No

Ferioli et al. [33]	3 v 3, 4 v 4	5 v 5	HR (% of maximal)	93.3 ± 2.3	92.5 ± 2.8	No
			RPE (AU)	6.6 ± 1.1	6.1 ± 1.1	Yes
			Low intensity activities (count)	12.0 ± 1.4	11.6 ± 1.7	No
			Moderate intensity activities (count)	4.5 ± 0.8	4.1 ± 1.0	No
			High intensity activities (count)	5.8 ± 1.0	5.3 ± 0.9	No
			Recovery (count)	6.9 ± 0.7	7.2 ± 0.6	No
			All movements (count)	29.3 ± 2.3	28.0 ± 3.3	No
			Assists (count)	3.5 ± 1.6	2.2 ± 1.1	Yes
			Personal founds (count)	0.9 ± 0.9	0.9 ± 0.8	No
			Rebounds (count)	5.4 ± 1.9	4.0 ± 1.4	Yes
			Steals (count)	2.4 ± 0.9	1.7 ± 1.3	Yes
			Turnovers (count)	3.8 ± 1.3	2.5 ± 1.6	No
			Total passes (count)	24.1 ± 5.4	19.4 ± 3.2	Yes
			Correct passes (count)	22.0 ± 4.9	17.7 ± 2.4	Yes
			Correct passes (%)	91.7 ± 6.4	92.1 ± 9.3	Yes
			Total shots (count)	9.4 ± 3.1	6.3 ± 2.2	Yes
			Scored shots (count)	4.1 ± 2.3	2.4 ± 1.4	Yes
			Scored shots (%)	41.1 ± 19.3	40.7 ± 24.8	Yes

Clemente et al. [34]	1 v 1, 2 v 2	3 v 3, 4 v 4	RPE (AU)	3.4 ± 1.1	3.7 ± 0.8	Yes
			Received balls (count • min^–1^)	1.6 ± 0.7	1.2 ± 0.4	Yes
			Conquered balls (count • min^–1^)	0.4 ± 0.4	0.1 ± 0.1	Yes
			Lost balls (count • min^–1^)	0.4 ± 0.4	0.2 ± 0.2	No
			Attacking balls (count • min^–1^)	0.8 ± 0.4	0.7 ± 0.4	Yes
			Shot (count • min^–1^)	1.4 ± 0.6	0.6 ± 0.3	Yes
			Rebound (count • min^–1^)	0.8 ± 0.5	0.4 ± 0.3	Yes
	3 v 3, 4 v 4	5 v 5	RPE (AU)	3.7 ± 0.8	5.8 ± 0.6	Yes
			Received balls (count • min^–1^)	1.2 ± 0.4	1.0 ± 0.4	Yes
			Conquered balls (count • min^–1^)	0.1 ± 0.1	0.1 ± 0.1	Yes
			Lost balls (count • min^–1^)	0.2 ± 0.2	0.1 ± 0.1	No
			Attacking balls (count • min^–1^)	0.7 ± 0.4	0.6 ± 0.2	Yes
			Shot (count • min^–1^)	0.6 ± 0.3	0.4 ± 0.2	Yes
			Rebound (count • min^–1^)	0.4 ± 0.3	0.3 ± 0.2	Yes
	1 v 1, 2 v 2	5 v 5	RPE (AU)	3.4 ± 1.1	5.8 ± 0.6	Yes
			Received balls	1.6 ± 0.7	1.0 ± 0.4	No
			Conquered balls	0.4 ± 0.4	0.1 ± 0.1	No
			Lost balls	0.4 ± 0.4	0.1 ± 0.1	No
			Attacking balls	0.8 ± 0.4	0.6 ± 0.2	No
			Shot	1.4 ± 0.6	0.4 ± 0.2	No
			Rebound	0.8 ± 0.5	0.3 ± 0.2	No

Vaquera et al. [35]	3 v 2	5 v 5	Maximal HR (bpm)	88.7 ± 5.8	92.0 ± 3.5	No
			Mean HR (% of maximal)	78.5 ± 7.5	91.2 ± 4.7	No
			RPE (AU)	8.2 ± 1.1	7.9 ± 1.5	No
	1 v 1, 2 v 2	5 v 5	Maximal HR (bpm)	91.5 ± 3.5	92.0 ± 3.5	No
			Mean HR (% of maximal)	81.3 ± 4.3	91.2 ± 4.7	No
			RPE (AU)	8.7 ± 0.7	7.9 ± 1.5	Yes

Conte et al. [36]	2 v 2	4 v 4	HR (% of maximal)	91.8 ± 3.0	89.7 ± 3.1	Yes
			RPE (AU)	9.3 ± 3.0	8.4 ± 0.9	Yes
			Dribble (count)	22.8 ± 5.8	13.5 ± 8.2	Yes
			Pass (count)	21.4 ± 5.0	19.2 ± 6.6	Yes
			Shot (count)	16.7 ± 7.2	5.6 ± 2.3	Yes
			Interception (count)	1.7 ± 1.1	1.4 ± 1.6	Yes
			Steal (count)	0.9 ± 0.9	0.9 ± 1.0	Yes
			Rebound (count)	7.4 ± 3.4	3.1 ± 1.9	Yes
			Turnover (count)	2.3 ± 1.7	2.1 ± 2.0	No

Herran et al. [43]	3 v 3	5 v 5	Total distance (m)	249.6 ± 32.8	249.6 ± 32.8	No
			Total distance (m • min^–1^)	49.9 ± 6.6	41.8 ± 7.2	No
			Player load	47.6 ± 7.4	34.8 ± 8.6	No
			Maximum velocity (m • s^–1^)	3.0 ± 0.4	2.8 ± 1.1	No
			Speed < 0.5 (m • s^–1^)	48.7 ± 31.6	70.6 ± 35.3	No
			Speed 0.5 to 1.0 (m • s^–1^)	59.2 ± 14.8	59.2 ± 13.3	No
			Speed 1.0 to 1.5 (m • s^–1^)	60.6 ± 19.9	38.0 ± 21.3	No
			Speed 1.5 to 2.0 (m • s^–1^)	43.4 ± 19.1	23.8 ± 16.6	No
			Speed > 2.0 (m • s^–1^)	37.8 ± 16.1	17.6 ± 15.6	No
			Deceleration < 1.5 (m • s–2)	8.6 ± 3.1	5.2 ± 3.5	No
			Deceleration -1.5 to -1.0 (m • s–2)	7.6 ± 2.5	5.0 ± 2.7	No
			Deceleration -1.0 to -0.5 (m • s–2)	15.9 ± 5.6	11.9 ± 5.3	No
			Deceleration -0.5 to 0.0 (m • s–2)	42.5 ± 10.0	34.6 ± 10.6	No
			Acceleration 0.0 to 0.5 (m • s–2)	103.5 ± 20.3	81.2 ± 18.6	No
			Acceleration 0.5 to 1.0 (m • s–2)	16.4 ± 4.8	11.5 ± 4.6	No
			Acceleration 1.0 to 1.5 (m • s–2)	8.6 ± 2.3	5.9 ± 2.6	No
			Acceleration > 1.5 (m • s–2)	14.6 ± 4.9	9.8 ± 5.4	No

Conte et al. [37]	2 v 2	4 v 4	Maximal HR (%): total time	87.1 ± 2.9	84.5 ± 4.0	Yes
			Maximal HR (%): excluding recovery	89.9 ± 3.1	87.3 ± 4.2	Yes
			Rate of perceived exertion (AU)	8.8 ± 0.9	7.7 ± 1.1	Yes
			Edwards’ TRIMP (AU)	54.1 ± 3.5	51.0 ± 5.1	No
			Dribble (count)	23.7 ± 6.1	14.4 ± 7.2	Yes
			Steal (count)	2.5 ± 1.9	2.1 ± 1.6	Yes
			Rebound (count)	7.0 ± 3.3	3.3 ± 2.1	Yes
			Turnover (count)	2.9 ± 2.0	2.5 ± 1.9	No
			Total pass (count)	21.9 ± 5.4	17.7 ± 6.7	Yes
			Correct pass (count)	19.8 ± 5.3	15.9 ± 6.2	Yes
			Wrong pass (count)	2.1 ± 1.7	1.9 ± 1.4	No
			Total shot (count)	16.0 ± 5.3	6.8 ± 3.0	Yes
			Scored shot (count)	6.8 ± 3.7	2.8 ± 2.1	Yes
			Missed shot (count)	9.1 ± 3.3	4.0 ± 1.9	No
			Made shot (%)	40.8 ± 15.8	37.4 ± 23.1	Yes

Garcia et al. [38]	3 v 3	4 v 4	HR high intensity (% maximal)^1^	53.2	60.1	No
			HR moderate intensity (% maximal)^2^	37.5	31.7	No

Klusemann et al. [45]	2 v 2	4 v 4	Stand/walk (count)	120 ± 18	125 ± 23	No
			Jog (count)	63 ± 11	66 ± 12	No
			Run (count)	35 ± 10	35 ± 10	No
			Sprint (count)	15 ± 5	11 ± 5	No
			Low shuffle (count)	39 ± 12	42 ± 10	No
			Medium shuffle (count)	72 ± 19	75 ± 17	No
			High shuffle (count)	13 ± 6	8 ± 4	No
			Jump (count)	26 ± 5	16 ± 6	No
			Total movements (count)	382 ± 52	378 ± 51	No
			Peak HR (% maximal)	92 ± 3	92 ± 3	Yes
			Mean HR (% maximal)	86 ± 4	83 ± 5	Yes
			Time spent zone 4 (%)^3^	55 ± 24	51 ± 20	Yes
			Time spent zone 5 (%)^4^	30 ± 31	22 ± 25	Yes
			Dribble (game • player–1)	20 ± 6	12 ± 5	Yes
			Pass (game • player–1)	19 ± 5	15 ± 5	Yes
			Close range shot (game • player–1)	8 ± 3	3 ± 2	Yes
			Mid-range shot (game • player–1)	4 ± 3	2 ± 2	Yes
			3-point shot	3 ± 3	2 ± 2	Yes
			Rebound	8 ± 3	5 ± 3	Yes
			Ball-screen	5 ± 4	3 ± 3	Yes
			Total elements	68 ± 12	43 ± 10	Yes

Tallir et al. [44]	3 v 3	4 v 4	Decision making (%)	80.0 ± 12.8	80.1 ± 10.5	No
			MSE efficiency (%)	84.0 ± 12.7	85.8 ± 5.8	No
			MSE efficacy (%)	76.7 ± 13.0	78.9 ± 7.0	No

Sampaio et al. [39]	3 v 3	4 v 4	Mean HR (bpm)	173.4 ± 8.3	164.7 ± 6.2	No
			RPE (AU)	3.0 ± 0.5	4.1 ± 0.8	No

1 High intensity (> 85%);

2 Moderate intensity (70–85%);

3 Time spent zone 4 (80–89%);

4 Time spent zone 5 (90–100%).

SSG (small-sided games); SD (standard deviation); RPE (rate of perceived exertion); HR (heart rate); AU (arbitrary units); bpm (beats per minute); EL (external load).

¶ Variables were expressed with median values.

### Meta-analysis

**FIG. 4:**
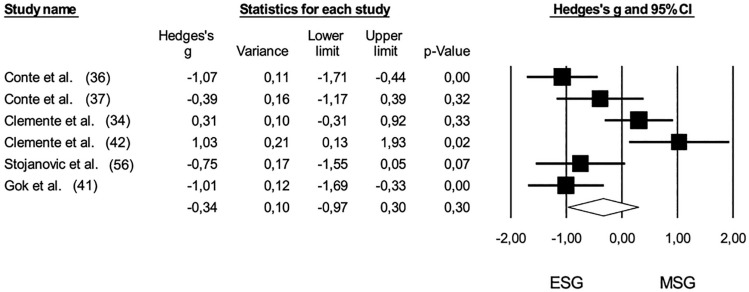
Forest plot of comparison in the rate of perceived exertion in basketball players participating in (ESG: 1 v 1; 2 v 2) and medium-sided games (MSG: 3 v 3; 4 v 4). The black diamond reflects the overall comparisons.

**FIG. 5:**
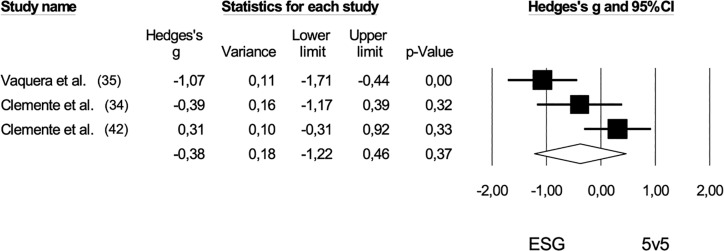
Forest plot of comparison in the rate of perceived exertion in basketball players participating in extreme-sided games (ESG: 1 v 1; 2 v 2) and 5 v 5. The black diamond reflects the overall comparisons.

**FIG. 6:**
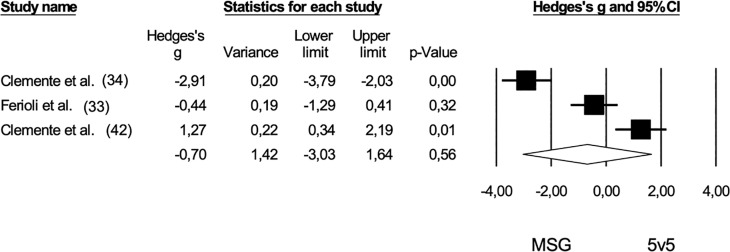
Forest plot of comparison in the rate of perceived exertion in basketball players participating in medium-sided games (MSG: 3 v 3; 4 v 4) and 5 v 5. The black diamond reflects the overall comparisons.

Six studies [32, 34, 36, 37, 41, 42] provided data on the rate of perceived exertion (RPE), comparing extreme small-sided games (ESG) and medium-sided games (MSG) with a pooled sample size of 93. The comparison of RPE between ESG and MSG showed no statistically significant difference (*Hedge’s g* = -0.34, 95% CI: -0.97 to 0.30; *p* = 0.30). Furthermore, no risk of publication bias was identified for this comparison (*Egger’s intercept* = 4.80; p = 0.52), and Duval and Tweedie’s correction did not reveal any trimmed studies (Figure 4). High levels of heterogeneity were observed (*I^2^* = 78%). When contrasting ESG and MSG with 5 v 5 regarding RPE, no significant differences were found among the game formats: ESG versus 5 v 5 (*pooled n* = 42, *Hedge’s g* = -0.38, 95% CI: -1.22 to 0.46; *p* = 0.37) and MSG versus 5 v 5 *(pooled n* = 40, *Hedge’s g* = -0.70, 95% CI: -3.03 to 1.64; p = 0.56). The negative effect sizes suggest higher RPE values in shorter game formats (Figures 5 and 6).

No publication bias was found for ESG compared to 5 v 5 (*Egger’s intercept* = -3.20; *p* = 0.88) and for MSG compared to 5 v 5 *(Egger’s intercept* = 49.83; *p* = 0.34). High heterogeneity values were also noted for both comparisons (ESG and 5 v 5: *I^2^* = 78%; MSG and 5 v 5: *I^2^* = 95%).

**FIG. 7:**
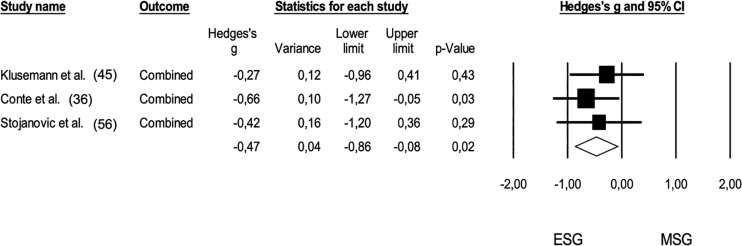
Forest plot of comparison in cardiovascular demands in basketball players participating in small-sided games (ESG: 1 v 1; 2 v 2) and medium-sided games (MSG: 3 v 3, 4 v 4). The black diamond reflects the overall comparisons. **Note:** The mean differences of heart rate outputs were converged in one effect size. The study of Klusemann et al. (45) combined the peak heart (% of maximal), mean heart rate (% of maximal), time spent in zone 4 (%), time spent in zone 3 (%); the study of Conte et al. (2015) converged maximal heart rate (% – total time and excluding recovery periods); the cardiovascular variables combined in the study of Stojanovic et al. (56) were mean heart rate (beats per minute), maximal heart rate (beats per minute), mean heart rate (% of maximal) and maximal heart rate (% of maximal).

In terms of cardiovascular responses, a meta-analysis was conducted to compare ESG and MSG, as shown in Figure 7 (pooled n = 49). The analysis revealed that cardiovascular demands were significantly higher during ESG (*Hedge’s g* = -0.47; 95% CI: -0.86 to -0.08; *p* = 0.02). The heterogeneity of this comparison was low (I^2^ < 5%), and no risk of publication bias was identified (*Egger’s intercept* = 2.83; *p* = 0.29). Additionally, two trimmed studies were identified, which resulted in an adjusted effect size that increased to a moderate level (*Hedge’s g* = -0.65; 95% CI: -0.96 to -0.34).

**FIG. 8:**
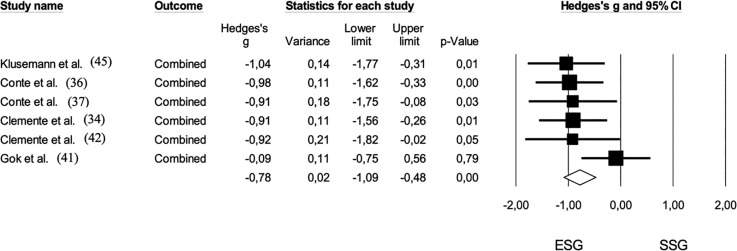
Forest plot of comparison in technical demands in basketball players participating in small-sided games (ESG: 1 v 1; 2 v 2) and medium-sided games (MSG: 3 v 3, 4 v 4). The black diamond reflects the overall comparisons. **Note:** The mean differences of heart rate outputs were converged in one effect size. The study of Klusemann et al. (45) combined the following variables: dribble, pass, close-range shot, mid-range shot, 3-point shot, rebound, ball screen, and total elements. The study of Conte et al. (37) combined the dribble, steal, rebound, total pass, correct pass, total shot, scored shot, made shot. Dribble, pass, shot, interception, steal, and rebound were converged in the study of Conte et al. (37). In the study of Clemente et al. (34), received balls, conquered balls, attacking balls, shots, and rebounds were converged. The study of Gok et al. (41) combined rebound, successful shot, successful pass, and steal.

The pooled sample size for the comparison between ESG and small-sided games on technical variables was 97 players. The effect size for this comparison was moderate, favoring ESG (*Hedge’s g* = -0.78; 95% CI: -1.09 to -0.48; *p* < 0.01). No trimmed studies were identified, and no risk of bias was observed (*Egger’s intercept* = -2.11; *p* = 0.29) (Figure 8).

**FIG. 9:**
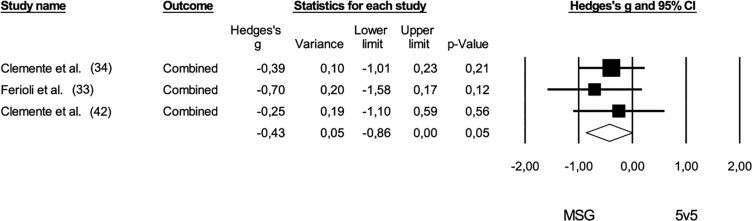
Forest plot of comparison in technical demands in basketball players participating in medium-sided games (MSG: 3 v 3; 4 v 4) and 5 v 5. The black diamond reflects the overall comparisons. **Note:** The study of Clemente et al. (34) combined received balls, conquered balls, attacking balls, shots, and rebounds. Assists, rebounds, steals, total passes, correct passes, total shots, and scored shots were converged in the study of Ferioli et al. (33). Received balls, conquered balls, attacking balls, shots, and rebounds were combined.

In the comparison between MSG and 5 v 5, the results tended to favor the shorter game format, showing a moderate effect size (*Hedge’s g* = -0.43; 95% CI: -0.86 to 0.00; *p* = 0.01). No trimmed studies were identified, and no risk of bias was noted (*Egger’s intercept* = -0.84; *p* = 0.39) (Figure 9). Heterogeneity across the studies was low in both meta-analyses, with *I^2^* values of 8% for ESG and MSG and less than 5% for MSG and 5 v 5.

## DISCUSSION

This systematic review with meta-analysis aimed to analyze the effects of different game formats (1 v 1, 2 v 2, 3 v 3, 4 v 4, 4 v 3, 3 v 3+1, and 5 v 5) on physiological, physical, technical, and tactical demands. The study findings revealed distinct increases across all four domains, with extreme SSG formats resulting in greater physiological and technical demands, while tactical and physical outcomes varied depending on game format and design.

### Physiological effects

The present meta-analysis revealed a significant impact on internal demands during ESG when compared to MSG and traditional 5 v 5 formats. This finding supports the hypothesis that ESG formats may elevate the physiological load, likely due to increased individual participation and more frequent high-intensity actions of play, such as rapid changes in direction, accelerations, and decelerations [16, 46, 47]. However, no significant differences were found in the RPE among the different SSG formats, which might suggest that while the physiological load increases, players do not necessarily perceive these games as more physically demanding. This could be due to the psychological aspect of gameplay, where engagement in smaller formats might mask physical exertion [48]. As highlighted by Delextrat and Martinez [48], basketball players may perceive a lower exertion level when they are more focused on ball interactions, decision-making, and tactical execution. The cognitive load associated with these tasks could potentially mask their perception of physical exertion, leading to a lower RPE despite high physiological demand [49]. Also, temporal factors, such as the time-point at which RPE is measured (mid-game, end-of-game, or post-game), could have contributed to the observed lack of differentiation [50].

However, the high heterogeneity observed indicates variability in how different studies measured or induced cardiovascular responses, possibly due to differences in game rules, player numbers, pitch size, or coaches’ profiles. The way game rules are structured can influence the intensity of gameplay; for example, changes in scoring systems, time constraints, or restrictions on player movement may result in differing levels of cardiovascular demand across formats [51]. Player numbers can also increase intensity—fewer players typically increase individual load, leading to more frequent high-intensity actions [52]. Also, smaller courts may lead to more frequent and intense short bursts of activity, while larger courts may require more sustained movement, thus affecting cardiovascular responses differently [53]. Finally, variations in coaches’ profiles, including coaching strategies, emphasis on fitness or tactical execution, and experience, could influence how players engage in the games, further contributing to differences in cardiovascular responses across studies [54].

Regarding the objective measures of cardiovascular demands in basketball players participating in ESG (1 v 1; 2 v 2) and MSG (3 v 3; 4 v 4), the present meta-analysis demonstrated that the objective measures of cardiovascular demands significantly impacted during ESG when compared to MSG. The significant impact on cardiovascular demands indicates that ESGs impose greater physiological stress on players compared to larger formats. This can be attributed to the structural differences in gameplay as fewer players on the pitch result in more individual involvement, reduced opportunities for passive recovery, and a need for continuous high-intensity movements [55]. A potential underlying mechanism for this impacted cardiovascular demand could be the relationship between external and internal loads. External load refers to the physical demands of the task, such as the intensity, frequency, and duration of movements, which are typically greater in ESGs due to the higher participation and more frequent high-intensity actions. Internal load, on the other hand, refers to the physiological responses to these external demands, such as heart rate, lactate accumulation, and perceived exertion. The higher external load in ESG formats likely leads to a greater internal load, which is reflected in the increased cardiovascular stress observed in these formats. These findings imply that ESGs could be particularly useful for improving aerobic capacity, which is vital for basketball players who need to maintain high levels of high-intensity activity throughout a basketball game. While ESGs can accelerate cardiovascular demands, they should be balanced with adequate recovery periods and possibly used in phases where high intensity is the main goal [56].

From a practical perspective, it is suggested that ESG, such as the 1 v 1 or 2 v 2 formats, are especially useful for improving cardiovascular fitness, as they expose basketball players to greater high-intensity actions, improving their aerobic power [17]. Given that basketball requires players to maintain high activity levels throughout a game, ESGs could be particularly beneficial during phases of training focused on cardiovascular development [57]. However, it is important to balance the use of ESGs with adequate recovery periods and other SSG formats to avoid excessive load and the accumulation of fatigue to optimize player performance over the long term [58].

### Technical effects

Considering the technical measures, the meta-analysis showed that ESG formats resulted in a significantly greater number of technical skills when compared to MSG and traditional 5 v 5 formats. This is likely due to more touches on the ball, increased involvement in the play, and the necessity for quicker decision-making [59]. However, the effect was more pronounced when comparing ESG with MSG than compared to traditional 5 v 5 formats. The increased frequency of technical actions in smaller SSG formats has implications for basketball training, particularly regarding skill acquisition. ESG, as demonstrated in this systematic review, creates an environment that demands more frequent technical actions, such as passing, dribbling, and shooting. This aligns with findings from multiple studies included in the review, where ESG (e.g., 1 v 1, 2 v 2) consistently had more frequent ball contact and technical involvements compared to larger formats such as the 5 v 5 format [33, 34, 36, 37, 41, 42, 45].

The impact on technical activity may be largely attributed to the reduced number of players per side, which increases each player’s participation in-game actions [60]. For instance, the study by Stojanovic et al. [32] revealed that in 1 v 1 and 2 v 2 formats, each player is engaged more frequently in offensive and defensive exchanges, resulting in more opportunities to practice decision-making under pressure. This forced interaction between players and the ball supports skill development by providing a higher number of task repetitions in short intervals [61]. Given that smaller formats allow for the continuous involvement of players, such SSG formats are more likely to improve technical proficiency. In the context of basketball, technical actions are integral to game success. In smaller SSGs, these actions are performed under more congested and pressured conditions, as players must execute their skills in smaller spaces and in less time [62]. This was particularly evident in two studies [31, 42] where smaller SSGs led to a greater number of total technical actions per minute compared to larger formats.

ESG challenges players to make faster decisions, improving their ability to anticipate and react to opponents’ movements, which is a crucial aspect of skill development in basketball [63]. Furthermore, ESG formats seem to provide a greater base for skill learning through variability [64]. Variability is an essential principle in skill acquisition, as it allows athletes to adapt their skills to different contexts and situations [61]. Another key factor in the increased technical involvement in smaller SSG formats is the pitch dimensions and the area per player. For example, studies such as those conducted by Sansone et al. [30] and Bredt et al. [31] showed that smaller pitch dimensions and fewer players resulted in a higher frequency of player involvement in both offensive and defensive actions. The area ratio per player illustrates how pitch size and player density influence technical behavior. For instance, in formats like 3 v 3, the area per player can be as low as 31.5 m^2^ [31], whereas in larger formats like 5 v 5, this increases to around 42 m^2^ [33]. This difference in playing space shows that players are under constant pressure in smaller SSG formats to perform more frequent and efficient technical actions.

### Tactical effects

Tactical behaviors were less frequently studied than physiological or technical variables. However, the available data suggests that ESG results in greater tactical involvement due to reduced team size and increased ball contact. In ESG, players are required to make quicker decisions under pressure, often in congested spaces, which likely augments tactical awareness and execution [65]. For example, two of the included studies in the present systematic review [32, 42] reported more frequent 1 v 1 and 2 v 2 interactions in smaller formats, where players were actively engaged in offensive and defensive strategies. Conversely, MSG and traditional 5 v 5 formats provide a broader range for team-oriented tactical behaviors, such as structured offensive and defensive plays, due to the increased player density and space available. This aligns with findings [33, 34], showing that tactical variables like spatial awareness and off-ball movements were more prevalent in larger formats. These findings suggest that while ESG augments individual tactical skills such as decision-making and quick reactions, MSG and 5 v 5 allow players to practice collective tactical systems, which are essential for game strategies.

### Study limitations and future research

Several limitations should be acknowledged. The limited number of included studies, especially those focused on ESG, limited our analyses. Although a comprehensive search was conducted, several studies were excluded due to insufficient data reporting or failure to meet eligibility criteria. Future studies should aim for greater standardization in reporting SSG formats, player demographics, and contextual variables to enable greater comparisons across different formats. The high heterogeneity observed in several measures, particularly in RPE and cardiovascular responses, limited us to draw consistent conclusions. Future research should establish more homogeneous experimental conditions. Furthermore, longitudinal studies and randomized controlled trials should be employed to address the identified gaps. Longitudinal studies would allow for the examination of the long-term effects of different SSG formats on player development and performance, while randomized controlled trials would provide more robust evidence regarding the causal effects of SSG formats on physiological, tactical, and technical outcomes. Lastly, this meta-analysis primarily focused on short-term physiological and technical responses during different SSGs. Future research should investigate the long-term effects of different playing formats on player development, including how exposure to different SSG formats influences physical and tactical performance over time.

### Practical Applications

Based on the findings of this meta-analysis, it is suggested that ESG, such as 1 v 1 and 2 v 2, be incorporated into basketball training to improve cardiovascular demands and technical proficiency. These formats may be effective in increasing the physiological load, improving aerobic power, and promoting higher levels of player involvement in technical actions. Coaches should also consider integrating ESG into phases of training focused on developing aerobic capacity/power and technical skills. Furthermore, programming the intensity and frequency of ESG sessions to match the specific needs of individual players or team strategies is of utmost importance. By varying training intensities and including both small-sided and other traditional formats, players can be exposed to different game situations, which contribute to the athletes’ preparedness across different aspects of the game.

## CONCLUSIONS

This systematic review with meta-analysis showed that ESGs resulted in higher cardiovascular demands and technical actions compared to MSGs, although no significant differences in RPE were found. These findings suggest that players may not perceive ESGs as more demanding despite their higher physiological load. The increased ball-related actions and decision-making in ESGs may mask exertion perceptions, making them effective for skill development and cardiovascular conditioning. Coaches should incorporate ESGs, such as 1 v 1 and 2 v 2 formats, to improve aerobic capacity, technical skills, and tactical actions. Overall, ESG formats can augment both physiological and technical demands in basketball training.
